# Essential Medicines at the National Level: The Global Asthma Network’s Essential Asthma Medicines Survey 2014

**DOI:** 10.3390/ijerph16040605

**Published:** 2019-02-19

**Authors:** Karen Bissell, Philippa Ellwood, Eamon Ellwood, Chen-Yuan Chiang, Guy B. Marks, Asma El Sony, Innes Asher, Nils Billo, Christophe Perrin, the Global Asthma Network Study Group

**Affiliations:** 1 School of Population Health, University of Auckland, Auckland 1023, New Zealand; 2 Department of Paediatrics: Child and Youth Health, University of Auckland, Auckland 1023, New Zealand; p.ellwood@auckland.ac.nz (P.E.); e.ellwood@auckland.ac.nz (E.E.); i.asher@auckland.ac.nz (I.A.); 3 Division of Pulmonary Medicine, Department of Internal Medicine, Wan Fang Hospital, Taipei Medical University, Taipei 116, Taiwan; cychiang@theunion.org; 4 Department of Internal Medicine, School of Medicine, College of Medicine, Taipei Medical University, Taipei 110, Taiwan; 5 South Western Sydney Clinical School, University of New South Wales, Sydney 2085, Australia; guy.marks@sydney.edu.au; 6 The Epidemiological Laboratory (Epi-Lab), for Public Health and Research, Khartoum, Sudan; asmaelsony@gmail.com; 7 Independent Consultant, 80220 Joensuu, Finland; nbillo@gmail.com; 8 Farmalex, 75008 Paris, France; kristofperrin@yahoo.fr

**Keywords:** essential medicines, access, noncommunicable diseases, asthma, inhaled corticosteroids, bronchodilators, national reimbursement list

## Abstract

Patients with asthma need uninterrupted supplies of affordable, quality-assured essential medicines. However, access in many low- and middle-income countries (LMICs) is limited. The World Health Organization (WHO) Non-Communicable Disease (NCD) Global Action Plan 2013–2020 sets an 80% target for essential NCD medicines’ availability. Poor access is partly due to medicines not being included on the national Essential Medicines Lists (EML) and/or National Reimbursement Lists (NRL) which guide the provision of free/subsidised medicines. We aimed to determine how many countries have essential asthma medicines on their EML and NRL, which essential asthma medicines, and whether surveys might monitor progress. A cross-sectional survey in 2013–2015 of Global Asthma Network principal investigators generated 111/120 (93%) responses—41 high-income countries and territories (HICs); 70 LMICs. Patients in HICs with NRL are best served (91% HICs included ICS (inhaled corticosteroids) and salbutamol). Patients in the 24 (34%) LMICs with no NRL and the 14 (30%) LMICs with an NRL, however no ICS are likely to have very poor access to affordable, quality-assured ICS. Many LMICs do not have essential asthma medicines on their EML or NRL. Technical guidance and advocacy for policy change is required. Improving access to these medicines will improve the health system’s capacity to address NCDs.

## 1. Introduction

Asthma, one of the major chronic diseases, has become a cause for global concern due to its increasing prevalence, morbidity, and economic impact. The Global Burden of Disease study estimated that there are 339 million people with asthma globally [1]. In 2016, it ranked 16th in the global causes of years lived with disability [2]. Since asthma is more prevalent among younger working age groups, it also has a major economic impact due to loss of productivity [3].

Patients with a chronic condition such as asthma need access to an uninterrupted supply of appropriate, effective, quality-assured essential medicines which need to be affordable for patients over the long term. However, studies continue to indicate a lack of availability and affordability of asthma essential medicines, especially in low- and middle-income countries (LMICs), and draw attention to medicine-related barriers to effective asthma management [4,5,6,7,8,9,10]. Serious concerns persist about the weakness of pharmaceutical quality assurance and of the capacity among national regulatory authorities in these countries [11].

Following the United Nations High Level Meeting on Noncommunicable Diseases (NCDs) in 2011, the World Health Organization developed a Global Action Plan for the Prevention and Control of Noncommunicable Diseases 2013–2020 (GAP) [12]. The GAP includes a number of voluntary targets, one of which relates to improving access to essential medicines. Countries are to achieve “an 80% availability of the affordable basic technologies and essential medicines, including generics, required to treat major NCDs in both public and private facilities” [12]. In many countries, access to NCD medicines is well below this target [13]. Calls have been made to implement systematic monitoring of progress in improving access to NCD medicines [14], including the tracking of national targets and indicators [15].

The World Health Organization (WHO) defines essential medicines as those that satisfy the priority health care needs of the population. Such medicines are intended to be available within the context of functioning health systems at all times in adequate amounts, in the appropriate dosage forms, with assured quality, and at a price that the individual and the community can afford. WHO promotes the concept of a national Essential Medicines List (EML), which is a government-approved selective list of medicines which guides the procurement and supply of medicines in the public sector, schemes that reimburse medicine costs, medicine donations, and local medicine production. When properly resourced, an EML is a cost-effective means of providing safe, effective treatment for the majority of high burden diseases [16]. However, availability is only part of the solution; affordability is also crucial. Cost to consumers is driven by the market price and the availability of subsidies or reimbursement. Where reimbursement is available, it is important that effective treatments for high burden diseases are included on the list of reimbursed medications. Thus, there are two national-level lists that countries can use to facilitate access to appropriate and affordable medicines: a national EML and a national reimbursement list (NRL).

For asthma, the 2013 version of the WHO EML, current at the time of the survey, included two types of inhaled corticosteroids (ICS): beclometasone 50 micrograms (µg) and 100 µg, and budesonide 100 µg and 200 µg, as well as one inhaled bronchodilator: salbutamol 100 µg as the medicines that are generally available at the lowest price, based on international drug price information sources [17]. These off-patent medicines are also included in the three global guidelines and manuals about asthma management, which all acknowledge the importance of prioritising cost-effective essential medicines for primary health care [18,19,20] and are still in the current WHO EML [21]. Since people with persistent asthma require long term treatment with both ICS and bronchodilator aerosols, EMLs should include items from both of these classes.

However, it is not clear how many countries include and/or update the essential asthma medicines on their EML and how many have asthma medicines on an NRL so that these medicines can be provided free or at subsidised prices to populations, especially the poor and vulnerable. Thus, the Global Asthma Network (GAN) undertook a survey to determine (a) how many countries have the essential asthma medicines on their EML and/or NRL, (b) which essential asthma medicines they include, and (c) whether surveys of an international network might help to monitor progress.

## 2. Methods

A cross-sectional email survey was conducted between 2013 and 2015. The questionnaire and cover letter were emailed by the GAN Global Centre (Auckland) to the principal investigator of each of 276 GAN centres in 120 countries and territories, including 41 high-income countries and territories (HICs) and 79 LMICs. Three French overseas territories were included as HICs since they have always been presented as distinct centres for asthma prevalence surveys that have been undertaken by the International Study on Asthma and Allergies in Childhood (ISAAC) [22] and GAN.

The survey asked two main questions. Respondents were asked whether their country’s EML and NRL contained each of three WHO EML asthma medicines in dosages listed in the WHO EML (beclometasone 50 µg and 100 µg, and budesonide 100 µg and 200 µg, and salbutamol 100 µg). If they knew their country had no such list, they were to write “Not applicable”. Otherwise, they were to write “Yes”, “No” or “Don’t know”.

Data were entered into an Excel spreadsheet. Questionnaire responses were checked and any unclear answers were queried with the respondents. Where multiple questionnaires with different answers were returned for the same country, the GAN Global Centre staff discussed via email with one or more of the principal investigators to determine the appropriate answer.

Countries were analysed in two categories: high-income and low- and middle-income, in accordance with World Bank classification 2014 [23]. Medicines were analysed as separate items (5), however the ICS were also analysed as the group “Any ICS” (4). Basic descriptive analysis was performed.

Ethics approval was not sought as this survey collects data that are routinely available and does not use any personal data. Principal investigators are mainly respiratory specialists who work in major health facilities in their country. Participation in GAN research is voluntary and is not remunerated.

## 3. Results

Data were returned for 111 (41 HICs and 70 LMICs) of 120 countries that were surveyed (93% response rate). Results for each country are presented in Table 1, Table 2, Table 3 and Table 4, with countries grouped as HICs (Table 1 and Table 3) and LMICs (Table 2 and Table 4). Table 5 provides a summary of the data.

### 3.1. Essential Medicine Lists

Of the 111 responding countries, 90 (81%) had an EML. A higher percentage of LMICs reported having an EML: 68 (97%) LMICs versus 22 (54%) HICs. However, of the 90 countries with an EML, only 71 (79%) included one or more inhaled corticosteroid. This included 18 (82%) HICs and 53 (78%) LICs. The proportion of all countries that included salbutamol on their EML was higher (87%) than the proportion that included any ICS (79%).

### 3.2. National Reimbursement Lists

Fewer countries had an NRL than an EML (73% versus 81%). Of the 81 (73%) countries that reported having an NRL, 65 (80%) had one or more ICS on the NRL. There was a marked difference between country income level regarding inclusion of one or more ICS on the NRL: 33 (94%) HICs versus 32 (70%) LMICs. Regarding dosages for each ICS, the income differences persisted: beclometasone 50 µg was included by 30 (86%) HICs versus 23 (50%) LMICs; beclometasone 100 µg was included by 29 (83%) HICs versus 20 (43%) LMICs. Only 16 (35%) LMICs had both dosages of beclometasone on their NRL versus 26 (74%) HICs. Best reimbursed was salbutamol: 70 (86%) countries included it; 94% of HICs and 80% of LMICs.

A large difference between country income groups was observed for the inclusion of any ICS and salbutamol on the NRL: 32 (91%) HICs and 31 (67%) LMICs with a *p* value of 0.01.

## 4. Discussion

This survey shows that many countries do not have all the WHO-recommended essential asthma medicines at the recommended dosages on their EML. Many country representatives also report that the medicines are not included in any type of NRL, which probably indicates that the medicines are not being provided free or subsidised for patients.

The concepts of essential medicines, of access to essential medicines as a human right, and universal coverage are relevant for all countries, regardless of income [14,24,25]. The national EML, a concept launched in 1977, has retained an important role in creating and maintaining access to medicines over the years [26]. In 2015, Sustainable Development Goal 3.8 was created with a target to achieve “access to safe, effective, quality and affordable essential medicines and vaccines for all” [27]. The WHO includes the EML as a key component of a well-functioning health system, noting that it is to guide procurement, reimbursement, and training [28]. It calls it “a guide for national committees that are responsible for identification of the most cost-effective medicines at country level for procurement, reimbursement, and treatment decisions, and helps to focus national efforts towards universal coverage”. Although the nature of the EML may be expanding slightly to incorporate recently released medicines that are still expensive, this does not contradict the basic principle of prioritising medicines from a clinical and public health perspective, and aiming to achieve access for all, especially the poor and vulnerable [29]. Getting medicines on the EML is still viewed as a first step towards access [29]. The WHO Director-General recently commented on access to new medicines: “[p]lacing them on the WHO Essential Medicines List is a first step in that direction” [30].

Health systems are dynamic, complex systems with barriers that can occur at multiple levels and be interrelated [31]. A lack of prioritisation of asthma medicines in national level documents, such as EMLs and NRLs, is likely to create policy- and supply-related barriers to access that, in turn, impact many levels and many other processes in the health system. With a cumulative effect, these barriers are likely to have a detrimental effect on patient pathways, access, and adherence to treatment.

The survey identified some major gaps in LMICs regarding the existence of national lists and the inclusion of medicines. Although LMICs had higher percentages than HICs for the existence of an EML and the inclusion of salbutamol on the EML, they had consistently lower percentages for all other questions.

The results relating to inclusion of ICS on national lists in LMICs are of particular concern. Firstly, 22% of LMICs with an EML still do not include any ICS, and 14 (30%) of those with an NRL do not include any ICS. In addition, it is likely that the 24 (34%) LMICs that reported not having an NRL are unlikely to have any other way of making ICS affordable for patients. Thus, patients are likely to find ICS unaffordable in 38 (54%) of the total LMICs surveyed.

Secondly, ICS, the preventer medicines that lead to improved asthma control, are less commonly reimbursed by LMIC governments than salbutamol, the short-term reliever inhaler that does not reduce the burden of asthma in the long term if applied without an ICS. This may reflect an ongoing misunderstanding among some policy-makers, specialist physicians, and other health workers about the importance of ICS in the long-term management of asthma. The lack of prioritisation of ICS in national level lists is likely to be playing a role in the underprescription and underuse of ICS which continues to be reported [32,33,34].

Thirdly, few LMICs have both dosages of beclometasone or budesonide. Yet, a range of doses of ICS is needed so that the appropriate and convenient dosage can be prescribed for each level of disease severity and for children as well as adults. Patient outcomes and adherence may be unfavourably affected by this constraint.

Most results for most HICs appear favourable, however they should be interpreted with some caution. Patients in the 91% of HICs reporting an NRL that include both ICS and salbutamol may have good access to the complete treatment that is required for their asthma. However, it would be incorrect to assume that including asthma medicines on an EML or an NRL means that they are in practice affordable and available for all groups of their populations [35]. Access for low-income and vulnerable populations in HICs may in fact be as poor as in some LMICs. A study in Denmark found that high levels of socio-economic status were positively associated with high use of ICS and concluded that attention should be paid to asthma sufferers with low educational level and low income [36].

In our survey, 18 HICs reported not having an EML, seven reported no NRL, and two reported having neither. It is a country level decision whether to formulate such national lists or not. However, if there are no national documents to guide decision-making, there is a risk that governments might lose sight of the principles behind essential medicines. A qualitative stakeholder analysis that interviewed medicine decision-makers in Australia found much variety in definitions and understanding about the concept of essential medicines and its application with regard to reimbursement decisions [37]. Researchers and practitioners in Canada have developed a preliminary list of essential medicines for primary care, in line with parliamentary recommendations and a concern that suboptimal prescribing might be partially related to the large number of medicines that are available [38,39].

There may be a number of reasons why countries have not included the recommended asthma medicines or dosages. Some EMLs and NRLs may not have been updated recently or since the changeover from chlorofluorocarbon-propelled inhalers, which saw the dosages change for beclometasone. It may be that there are no national asthma guidelines, meaning that individual specialists and health services select medicines unguided; a low priority for asthma and chronic respiratory diseases in general [40,41]; or strong undue influence on the EML selection process due to pharmaceutical or trade-related lobbying. It may be that countries have other dosages or formulations of these medicines, or other asthma medicines in circulation. Other medicines are likely to be considerably more expensive and thus less accessible, especially for poorer populations.

A number of major limitations should be noted. The survey relied on self-reported data sent by a single individual. It is possible that principal investigators might have incorrectly interpreted information about their national lists. Within the scope of this unfunded survey, however, it would have been impractical for GAN to request copies of EMLs and lists of reimbursed medicines in an attempt to validate responses. In addition to time and cost considerations, country lists are written in the language of the country and are complex to interpret without knowledge of each country’s health system. Since the principal investigators are researchers and practitioners who are familiar with the medicines involved, we believed that the risk of respondent error would been low.

A further limitation is that the survey did not explore any health systems’ details about the context, nature, or date of the latest update of the EMLs or NRLs. It is likely to have been difficult for some countries, especially federal and decentralised systems, to answer about the existence of a national reimbursement list or similar. Some respondents asked for clarification of our definition of NRL, however might have felt their answer was not representative of their country’s health system. Thus, the results may be misrepresenting the policy situation in some countries and they should be viewed with some caution. Future surveys could develop the questions about reimbursed and free medicines, expand guidance and explanations, and include a method for validating responses. 

The survey has several strengths. It was an inexpensive, rapid way to gain a snapshot of the inclusion on national level lists of essential asthma medicines in a large number of countries (111) from all WHO regions, including a large number of LMICs (70). The response rate of 93% was high for a survey, perhaps reflecting the longstanding collaboration between many principal investigators and those involved in GAN, the conciseness of the survey, and the importance accorded to the topic of access to essential medicines by principal investigators.

The survey highlights areas for action. Firstly, the monitoring of progress towards NCD medicine availability targets is important as it can identify gaps. Roles and resources for monitoring should be allocated at sub-national, national, and global levels so that various entities can contribute to improving medicine availability. We support calls for systematic monitoring of progress that is built into national routine information systems and includes policy and health systems indicators [42,43]. Alongside efforts to set up these important national systems, which may take some time, surveys could also be undertaken by global networks, such as GAN, comprised of committed health professionals who are used to applying standardised protocols [44] and the Global Alliance for Chronic Respiratory Disease. At a minimum, there should be regular surveys of availability, pricing, and affordability in public and private health facilities of the essential asthma medicines, for example, following the WHO-HAI methodology [45]. Spacers, which enable a subset of patients, in particular children, to use ICS inhalers effectively, could be included.

Secondly, some countries may want to update their lists and work out whether their other national level policies, strategies, and procedures are aligned to facilitate access to asthma essential medicines. The Lancet Commission on Essential Medicines Policies has provided a comprehensive series of actionable recommendations related to financing, affordability, quality and safety, quality use of medicines, developing missing medicines, and measuring progress [43]. These should help countries to take concrete steps towards achieving the 80% NCD medicine availability target [12] and Sustainable Development Goal target 3.8 [27]. Human and financial resources will need to be allocated for the necessary technical and policy work. International entities that promote access to essential medicines should see how they can assist where requested.

Thirdly, this survey should stimulate enquiry about the transparency of the medicine selection process at the national level and why ICS, in particular, are still missing from so many EMLs and NRLs. Attention should be drawn to the fact that undue influence over the selection processes may stem from conflicts of interests from governments, donor organisations, pharmaceutical companies, among other entities. We recommend advocating for national level medicines lists and policies that will promote evidence-based decision-making. Communities could take a rights-based approach to access to essential medicines and track progress towards the 80% availability target, holding governments accountable for gaps in access to NCD medicines, such as those for asthma [14]. We encourage all governments to reflect the principles of essential medicines and universal coverage in their medicines and reimbursement lists.

## 5. Conclusions

Many countries do not have the WHO-recommended essential asthma medicines on their national level medicine lists, and many are not providing the medicines free or subsidised for patients, especially in LMICs. This situation creates barriers for patient access to medicines and is likely to be perpetuating the underprescription and underuse of ICSs. One step towards the WHO 80% availability target for NCD essential medicines will be for countries to ensure that asthma essential medicines feature on their national medicines lists, with the correct dosages. Our survey obtained a very high response rate. With a strengthened methodology, international surveys could be one way to monitor progress towards the inclusion of essential asthma medicines on national level medicine lists. Progress should be monitored and evaluated at the national and international level. As part of the broader efforts to strengthen health systems, these policy-related measures are crucial first steps towards making these essential medicines available, quality-assured, and affordable for all.

## Figures and Tables

**Table 1:** Existence of National Medicine List and National Reimbursement List and inclusion of any inhaled corticosteroid and salbutamol, 41 high-income countries and territories, 2014 *.

High-Income Countries and Territories	Have EML	Any ICS on EML	S on EML	Have NRL	Any ICS on NRL	S on NRL
Australia	N	n/a	n/a	Y	Y	Y
Austria	N	n/a	n/a	Y	Y	Y
Belgium	Y	N	Y	Y	Y	Y
Canada	Y	Y	Y	Y	Y	Y
Channel Islands	Y	Y	Y	Y	Y	Y
Chile	Y	Y	Y	Y	Y	Y
Croatia	Y	Y	Y	Y	Y	Y
Cyprus	N	n/a	n/a	Y	Y	Y
Denmark	Y	Y	N	Y	N	N
Faroe Islands	Y	Y	Y	Y	Y	Y
Finland	N	n/a	n/a	Y	Y	Y
France	N	n/a	n/a	Y	Y	Y
French Polynesia	N	n/a	n/a	Y	Y	Y
Germany	N	n/a	n/a	Y	Y	Y
Hong Kong	N	n/a	n/a	Y	Y	Y
Ireland	N	n/a	n/a	Y	Y	Y
Israel	Y	Y	N	Y	Y	N
Italy	Y	Y	Y	Y	Y	Y
Japan	N	n/a	n/a	Y	Y	Y
Korea, South	Y	Y	Y	N	n/a	n/a
Kuwait	Y	Y	Y	Y	Y	Y
Latvia	Y	N	Y	Y	N	Y
Malta	N	n/a	n/a	Y	Y	Y
Netherlands	N	n/a	n/a	Y	Y	Y
New Caledonia	N	n/a	n/a	Y	Y	Y
New Zealand	N	n/a	n/a	Y	Y	Y
Norway	N	n/a	n/a	Y	Y	Y
Oman	Y	Y	Y	N	n/a	n/a
Poland	Y	Y	Y	Y	Y	Y
Portugal	Y	N	N	Y	Y	Y
Reunion Island	N	n/a	n/a	Y	Y	Y
Russia	Y	Y	Y	Y	Y	Y
Saudi Arabia	Y	Y	Y	N	n/a	n/a
Singapore	Y	Y	Y	Y	Y	Y
Spain	N	n/a	n/a	Y	Y	Y
Trinidad and Tobago	Y	Y	Y	Y	Y	Y
United Arab Emirates	Y	Y	Y	Y	Y	Y
United Kingdom	Y	Y	Y	Y	Y	Y
United States	N	n/a	n/a	N	n/a	n/a
Uruguay	Y	N	Y	N	n/a	n/a
Vatican City	N	n/a	n/a	N	n/a	n/a
**Total Y/total HICs (41)**	22			35		
**Total Y/total HICs with EML (22)**		18	19			
**Total Y/total HICs with NRL (35)**					33	33
**Total as %**	54	82	86	85	94	94

* N: No; n/a: not applicable; Y: Yes. Where a list does not exist, medicine data are marked as n/a. EML: Essential Medicine List; ICS: Inhaled corticosteroid; HICs: High-income countries and territories; NRL: National Reimbursement List; S: Salbutamol.

**Table 2:** Existence of National Medicine List and National Reimbursement List and inclusion of any inhaled corticosteroid and salbutamol, 70 low- and middle-income countries, 2014 *.

Low- and Middle-Income Countries	Have EML	Any ICS on EML	S on EML	Have NRL	Any ICS on NRL	S on NRL
Albania	Y	Y	Y	Y	Y	Y
Algeria	Y	Y	Y	Y	Y	Y
Argentina	Y	N	N	Y	Y	Y
Armenia	Y	N	Y	Y	N	Y
Belarus	Y	Y	Y	Y	Y	Y
Benin	Y	N	N	N	n/a	n/a
Bolivia	Y	Y	Y	Y	N	Y
Bosnia and Herzegovina	Y	N	Y	Y	N	Y
Brasil	Y	Y	Y	Y	N	N
Bulgaria	Y	Y	Y	Y	Y	Y
Burkina Faso	Y	Y	Y	Y	N	N
Cameroon	Y	N	N	N	n/a	n/a
China	Y	Y	Y	Y	Y	Y
Colombia	Y	Y	Y	N	n/a	n/a
Congo Dem Rep	Y	Y	Y	N	n/a	n/a
Costa Rica	Y	Y	Y	Y	Y	Y
Ecuador	Y	Y	Y	Y	Y	Y
Egypt	Y	N	N	Y	N	N
El Salvador	Y	Y	Y	Y	Y	Y
Fiji	Y	Y	Y	Y	Y	N
Gambia	Y	N	N	N	n/a	n/a
Georgia	Y	N	N	N	n/a	n/a
Ghana	Y	Y	Y	Y	Y	Y
Grenada	Y	Y	Y	N	n/a	n/a
Hungary	Y	Y	Y	Y	Y	Y
India	Y	Y	Y	Y	Y	Y
Indonesia	Y	Y	Y	Y	N	Y
Iran	Y	Y	Y	Y	Y	Y
Jamaica	Y	Y	Y	Y	Y	Y
Jordan	Y	Y	Y	Y	Y	Y
Kenya	Y	N	N	N	n/a	n/a
Kosovo	Y	N	Y	Y	N	N
Lao PDR	Y	N	Y	Y	N	N
Macedonia	Y	Y	Y	Y	Y	Y
Malawi	Y	Y	Y	N	n/a	n/a
Malaysia	Y	Y	Y	N	n/a	n/a
Mexico	Y	Y	Y	Y	Y	Y
Mongolia	Y	Y	Y	Y	N	Y
Nicaragua	Y	Y	Y	Y	Y	Y
Nigeria	Y	Y	Y	N	n/a	n/a
Niue	Y	Y	Y	N	n/a	n/a
Pakistan	Y	N	N	N	n/a	n/a
Palau	Y	Y	Y	N	n/a	n/a
Palestine	Y	Y	Y	N	n/a	n/a
Panama	Y	Y	Y	Y	Y	Y
Peru	Y	Y	Y	Y	Y	Y
Philippines	Y	Y	Y	Y	Y	Y
Romania	Y	Y	Y	Y	Y	Y
Samoa	Y	Y	Y	Y	N	N
Senegal	Y	N	N	Y	N	N
Serbia	Y	Y	Y	Y	Y	Y
Sierra Leone	Y	Y	Y	N	n/a	n/a
South Africa	Y	Y	Y	Y	Y	Y
Sri Lanka	Y	Y	Y	N	n/a	n/a
Sudan	Y	Y	Y	Y	Y	Y
Syrian Arab Republic	Y	N	Y	Y	Y	Y
Taiwan	N	n/a	n/a	Y	Y	Y
Thailand	Y	Y	Y	Y	Y	Y
Togo	Y	Y	Y	Y	Y	Y
Tokelau	Y	Y	Y	N	n/a	n/a
Tonga	Y	Y	Y	N	n/a	n/a
Tunisia	Y	Y	Y	Y	Y	Y
Turkey	N	n/a	n/a	Y	Y	Y
Tuvalu	Y	Y	Y	N	n/a	n/a
Uganda	Y	Y	Y	N	n/a	n/a
Ukraine	Y	Y	Y	Y	N	N
Vanuatu	Y	Y	Y	N	n/a	n/a
Vietnam	Y	N	Y	Y	N	Y
Zambia	Y	Y	Y	N	n/a	n/a
Zimbabwe	Y	Y	Y	N	n/a	n/a
**Total Y/total LMICs (70)**	68			46		
**Total Y/total LMICs with EML (68)**		53	59			
**Total Y/total LMICs with NRL (46)**					32	37
**Total as %**	97	78	87	66	70	80

* N: No; n/a: not applicable; Y: Yes. Where a list does not exist, medicine data are marked as n/a. EML: Essential Medicine List; ICS: Inhaled Corticosteroid; LMICs: Low- and middle-income countries; NRL: National Reimbursement List; S: Salbutamol.

**Table 3:** Inclusion of HFA inhalers from the WHO Essential Medicines List in National Essential Medicine and National Reimbursement Lists in 41 high-income countries and territories, 2014 *.

High-Income Countries and Territories	Beclometasone 50 μg		Beclometasone 100 μg		Budesonide 100 μg		Budesonide 200 μg		Salbutamol 100 μg	
	EML	NRL	EML	NRL	EML	NRL	EML	NRL	EML	NRL
Australia	n/a	Y	n/a	Y	n/a	N	n/a	N	n/a	Y
Austria	n/a	Y	n/a	Y	n/a	Y	n/a	Y	n/a	Y
Belgium	N	Y	N	N	N	N	N	Y	Y	Y
Canada	Y	Y	Y	Y	N	N	N	N	Y	Y
Channel Islands	Y	Y	Y	Y	Y	Y	Y	Y	Y	Y
Chile	Y	Y	Y	Y	Y	Y	Y	Y	Y	Y
Croatia	Y	Y	Y	Y	N	N	N	N	Y	Y
Cyprus	n/a	N	n/a	Y	n/a	N	n/a	N	n/a	Y
Denmark	N	N	Y	N	N	N	Y	N	N	N
Faroe Islands	Y	Y	Y	Y	Y	Y	Y	Y	Y	Y
Finland	n/a	Y	n/a	Y	n/a	N	n/a	N	n/a	Y
France	n/a	Y	n/a	Y	n/a	Y	n/a	Y	n/a	Y
French Polynesia	n/a	Y	n/a	Y	n/a	Y	n/a	Y	n/a	Y
Germany	n/a	Y	n/a	Y	n/a	Y	n/a	Y	n/a	Y
Hong Kong	n/a	Y	n/a	Y	n/a	Y	n/a	Y	n/a	Y
Ireland	n/a	Y	n/a	Y	n/a	N	n/a	N	n/a	Y
Israel	Y	Y	Y	Y	Y	Y	Y	Y	N	N
Italy	Y	Y	Y	Y	Y	Y	Y	Y	Y	Y
Japan	n/a	Y	n/a	Y	n/a	Y	n/a	Y	n/a	Y
Korea, South	Y	n/a	Y	n/a	Y	n/a	Y	n/a	Y	n/a
Kuwait	Y	Y	Y	Y	Y	Y	Y	Y	Y	Y
Latvia	N	N	N	N	N	N	N	N	Y	Y
Malta	n/a	Y	n/a	N	n/a	N	n/a	Y	n/a	Y
Netherlands	n/a	Y	n/a	Y	n/a	Y	n/a	Y	n/a	Y
New Caledonia	n/a	Y	n/a	Y	n/a	Y	n/a	Y	n/a	Y
New Zealand	n/a	Y	n/a	Y	n/a	N	n/a	N	n/a	Y
Norway	n/a	Y	n/a	Y	n/a	N	n/a	N	n/a	Y
Oman	Y	n/a	Y	n/a	N	n/a	N	n/a	Y	n/a
Poland	N	N	Y	Y	N	N	Y	Y	Y	Y
Portugal	N	Y	N	Y	N	Y	N	Y	N	Y
Reunion Island	n/a	Y	n/a	Y	n/a	Y	n/a	Y	n/a	Y
Russia	Y	Y	Y	Y	N	N	N	N	Y	Y
Saudi Arabia	Y	n/a	N	n/a	N	n/a	Y	n/a	Y	n/a
Singapore	Y	Y	N	N	N	N	N	N	Y	Y
Spain	n/a	Y	n/a	N	n/a	Y	n/a	Y	n/a	Y
Trinidad and Tobago	Y	Y	Y	Y	Y	N	Y	N	Y	Y
United Arab Emirates	N	N	Y	Y	Y	Y	Y	Y	Y	Y
United Kingdom	Y	Y	Y	Y	N	N	N	N	Y	Y
United States	n/a	n/a	n/a	n/a	n/a	n/a	n/a	n/a	n/a	n/a
Uruguay	N	n/a	N	n/a	N	n/a	N	n/a	Y	n/a
Vatican City	n/a	n/a	n/a	n/a	n/a	n/a	n/a	n/a	n/a	n/a
**Total Y/total HICs with EML (22)**	15		16		9		12		19	
**Total Y/total HICs with NRL (35)**		30		29		18		21		33
**Total as %**	68	86	73	83	41	51	55	60	86	94

* N: No; n/a: not applicable; Y: Yes. Where a list does not exist, medicine data are marked as n/a. WHO: The World Health Organization; EML: Essential Medicine List; HFA: hydrofluoroalkane; HICs: High-income countries and territories; NRL: National Reimbursement List.

**Table 4:** Inclusion of HFA inhalers from the WHO Essential Medicines List in National Essential Medicine and National Reimbursement Lists in 70 low- and middle-income countries, 2014 *.

Low- and Middle-Income Countries	Beclometasone 50 μg		Beclometasone 100 μg		Budesonide 100 μg		Budesonide 200 μg		Salbutamol 100 μg	
	EML	NRL	EML	NRL	EML	NRL	EML	NRL	EML	NRL
Albania	Y	Y	Y	Y	Y	Y	Y	Y	Y	Y
Algeria	N	N	N	N	Y	Y	Y	Y	Y	Y
Argentina	N	N	N	N	N	Y	N	Y	N	Y
Armenia	N	N	N	N	N	N	N	N	Y	Y
Belarus	Y	Y	Y	Y	Y	Y	Y	Y	Y	Y
Benin	N	n/a	N	n/a	N	n/a	N	n/a	N	n/a
Bolivia	Y	N	Y	N	Y	N	Y	N	Y	Y
Bosnia and Herzegovina	N	N	N	N	N	N	N	N	Y	Y
Brasil	Y	N	N	N	N	N	N	N	Y	N
Bulgaria	Y	Y	Y	Y	Y	Y	Y	Y	Y	Y
Burkina Faso	Y	N	N	N	N	N	Y	N	Y	N
Cameroon	N	n/a	N	n/a	N	n/a	N	n/a	N	n/a
China	Y	Y	N	N	N	N	N	N	Y	Y
Colombia	Y	n/a	N	n/a	N	n/a	N	n/a	Y	n/a
Congo Dem Rep	N	n/a	Y	n/a	N	n/a	N	n/a	Y	n/a
Costa Rica	N	N	Y	Y	N	N	N	N	Y	Y
Ecuador	Y	Y	Y	Y	N	N	N	N	Y	Y
Egypt	N	N	N	N	N	N	N	N	N	N
El Salvador	Y	Y	N	N	N	N	N	N	Y	Y
Fiji	N	N	Y	Y	N	N	N	N	Y	N
Gambia	N	n/a	N	n/a	N	n/a	N	n/a	N	n/a
Georgia	N	n/a	N	n/a	N	n/a	N	n/a	N	n/a
Ghana	N	N	Y	Y	Y	Y	Y	Y	Y	Y
Grenada	Y	n/a	Y	n/a	N	n/a	N	n/a	Y	n/a
Hungary	N	N	N	N	Y	Y	Y	Y	Y	Y
India	Y	Y	Y	Y	Y	Y	Y	Y	Y	Y
Indonesia	N	N	N	N	Y	N	Y	N	Y	Y
Iran	Y	Y	N	N	Y	Y	Y	Y	Y	Y
Jamaica	Y	Y	Y	Y	N	N	Y	Y	Y	Y
Jordan	Y	Y	Y	Y	N	N	N	N	Y	Y
Kenya	N	n/a	N	n/a	N	n/a	N	n/a	N	n/a
Kosovo	N	N	N	N	N	N	N	N	Y	N
Lao PDR	N	N	N	N	N	N	N	N	Y	N
Macedonia	Y	Y	N	N	N	N	Y	Y	Y	Y
Malawi	Y	n/a	Y	n/a	N	n/a	N	n/a	Y	n/a
Malaysia	N	n/a	Y	n/a	Y	n/a	Y	n/a	Y	n/a
Mexico	N	Y	N	Y	Y	Y	Y	Y	Y	Y
Mongolia	Y	N	Y	N	N	N	N	N	Y	Y
Nicaragua	Y	Y	Y	Y	N	N	N	N	Y	Y
Nigeria	Y	n/a	N	n/a	N	n/a	N	n/a	Y	n/a
Niue	N	n/a	Y	n/a	N	n/a	N	n/a	Y	n/a
Pakistan	N	n/a	N	n/a	N	n/a	N	n/a	N	n/a
Palau	N	n/a	Y	n/a	N	n/a	N	n/a	Y	n/a
Palestine	N	n/a	N	n/a	N	n/a	Y	n/a	Y	n/a
Panama	Y	Y	N	N	N	N	Y	Y	Y	Y
Peru	Y	Y	N	N	N	N	Y	Y	Y	Y
Philippines	Y	Y	Y	Y	N	N	Y	Y	Y	Y
Romania	Y	Y	Y	Y	Y	Y	Y	Y	Y	Y
Samoa	N	N	Y	N	N	N	N	N	Y	N
Senegal	N	N	N	N	N	N	N	N	N	N
Serbia	Y	N	Y	Y	Y	N	Y	Y	Y	Y
Sierra Leone	Y	n/a	Y	n/a	N	n/a	N	n/a	Y	n/a
South Africa	Y	Y	Y	Y	Y	Y	Y	Y	Y	Y
Sri Lanka	Y	n/a	Y	n/a	N	n/a	N	n/a	Y	n/a
Sudan	Y	N	N	N	N	N	N	Y	Y	Y
Syrian Arab Republic	N	Y	N	Y	N	Y	N	Y	Y	Y
Taiwan	n/a	N	n/a	N	n/a	N	n/a	Y	n/a	Y
Thailand	Y	Y	Y	Y	Y	Y	Y	Y	Y	Y
Togo	Y	Y	Y	Y	N	N	N	N	Y	Y
Tokelau	Y	n/a	N	n/a	N	n/a	N	n/a	Y	n/a
Tonga	N	n/a	Y	n/a	N	n/a	N	n/a	Y	n/a
Tunisia	Y	Y	N	N	N	N	N	Y	Y	Y
Turkey	n/a	Y	n/a	Y	n/a	Y	n/a	Y	n/a	Y
Tuvalu	Y	n/a	Y	n/a	N	n/a	N	n/a	Y	n/a
Uganda	Y	n/a	N	n/a	N	n/a	N	n/a	Y	n/a
Ukraine	N	N	N	N	Y	N	Y	N	Y	N
Vanuatu	Y	n/a	N	n/a	N	n/a	N	n/a	Y	n/a
Vietnam	N	N	N	N	N	N	N	N	Y	Y
Zambia	Y	n/a	N	n/a	N	n/a	N	n/a	Y	n/a
Zimbabwe	Y	n/a	Y	n/a	N	n/a	N	n/a	Y	n/a
**Total Y/total LMICs with EML (68)**	38		31		17		24		59	
**Total Y/total LMICs with NRL (46)**		23		20		15		24		37
**Total as %**	56	50	46	43	25	33	35	52	87	80

* N: No; n/a: not applicable; Y: Yes. Where a list does not exist, medicine data are marked as n/a. EML: Essential Medicine List; HFA: hydrofluoroalkane; LMICs: Low- and middle-income countries; NRL: National Reimbursement List.

**Table 5:** Existence of an Essential Medicine List and National Reimbursement List, and inclusion.

	High-Income Countries and Territories (41) N (%)	Low- and Middle-Income Countries (70) N (%)	Total N (%)	*p*-Value *
**Essential Medicines List (EML)**				
Have EML	22 (54)	68 (97)	90 (81)	<0.001
Any inhaled corticosteroid (ICS)	18 (82)	53 (78)	71 (79)	0.7
Salbutamol	19 (86)	59 (87)	78 (87)	0.9
Beclometasone 50 µg	15 (68)	38 (56)	53 (59)	0.3
Beclometasone 100 µg	16 (73)	31 (46)	47 (52)	0.03
Budesonide 100 µg	9 (41)	17 (25)	26 (29)	0.2
Budesonide 200 µg	12 (55)	24 (35)	36 (40)	0.1
**National Reimbursement List (NRL)**				
Have NRL	35 (85)	46 (66)	81 (73)	0.02
Any ICS	33 (94)	32 (70)	65 (80)	0.006
Salbutamol	33 (94)	37 (80)	70 (86)	0.07
Beclometasone 50 µg	30 (86)	23 (50)	53 (65)	<0.001
Beclometasone 100 µg	29 (83)	20 (43)	49 (60)	<0.001
Budesonide 100 µg	18 (51)	15 (33)	33 (41)	0.9
Budesonide 200 µg	21 (60)	24 (52)	45 (56)	0.5
Any ICS and Salbutamol	32 (91)	31 (67)	63 (78)	0.01

* *p* Value difference in proportion between high-income countries and territories and low- and middle-income countries.

## References

[1] GBD 2016 Causes of Death Collaborators (2017). Global, regional, and national age-sex specific mortality for 264 causes of death, 1980–2016: A systematic analysis for the Global Burden of Disease Study 2016. Lancet.

[2] GBD 2016 Disease and Injury Incidence and Prevalence Collaborators (2017). Global, regional, and national incidence, prevalence, and years lived with disability for 328 diseases and injuries for 195 countries, 1990–2016: A systematic analysis for the Global Burden of Disease Study 2016. Lancet.

[3] FitzGerald J.M., Sadatsafavi M. (2014). The importance of measuring asthma control in emerging economies. Int. J. Tuberc. Lung Dis..

[4] Babar Z.U., Lessing C., Macé C., Bissell K. (2013). The availability, pricing and affordability of three essential asthma medicines in 52 low- and middle-income countries. Pharmacoeconomics.

[5] Cameron A., Ewen M., Ross-Degnan D., Ball D., Laing R. (2009). Medicine prices, availability, and affordability in 36 developing and middle-income countries: A secondary analysis. Lancet.

[6] Cameron A., Roubos I., Ewen M., Mantel-Teeuwisse A.K., Leufkens H.G., Laing R.O. (2011). Differences in the availability of medicines for chronic and acute conditions in the public and private sectors of developing countries. Bull. World Health Organ..

[7] Zar H.J., Levin M.E. (2012). Challenges in treating pediatric asthma in developing countries. Paediatr. Drugs.

[8] Beran D., Zar H.J., Perrin C., Menezes A.M., Burney P., Forum of International Respiratory Societies working group c (2015). Burden of asthma and chronic obstructive pulmonary disease and access to essential medicines in low-income and middle-income countries. Lancet Respir. Med..

[9] Bissell K., Perrin C., Beran D. (2016). Access to essential medicines to treat chronic respiratory disease in low-income countries. Int. J. Tuberc. Lung Dis..

[10] Bissell K., Perrin C. (2014). Access to affordable, quality-assured asthma medicines. Global Asthma Report.

[11] Ravinetto R., Vandenbergh D., Macé C., Pouget C., Renchon B., Rigal J., Schiavetti B., Caudron J.-M. (2016). Fighting poor-quality medicines in low- and middle-income countries: The importance of advocacy and pedagogy. J. Pharm. Policy Pract..

[12] World Health Organization (2013). Global Action Plan for the Prevention and Control of Noncommunicable Diseases 2013–2020.

[13] World Health Organization (2014). Global Status Report on Noncommunicable Diseases.

[14] Hogerzeil H.V., Liberman J., Wirtz V.J., Kishore S.P., Selvaraj S., Kiddell-Monroe R., Mwangi-Powell F.N., Von Schoen-Angerer T., Selvaraj S. (2013). Promotion of access to essential medicines for non-communicable diseases: Practical implications of the UN political declaration. Lancet.

[15] World Health Organization (2016). Assessing National Capacity for the Prevention and Control of Noncommunicable Diseases: Report of the 2015 Global Survey.

[16] World Health Organization (2011). The World Medicines Situation 2011—Medicine Expenditures.

[17] World Health Organization (2013). 18th Model List of Essential Medicines.

[18] World Health Organization (2013). Package of Essential Noncommunicable (PEN) Disease Interventions for Primary Health Care in Low-Resource Settings.

[19] Ait-Khaled N., Enarson D.A., Chiang C.-Y., Marks G., Bissell K. (2008). Management of Asthma: A Guide to the Essentials of Good Clinical Practice.

[20] Global Initiative for Asthma (2018). Global Strategy for Asthma Management and Prevention, 2018.

[21] World Health Organization (2017). 20th Model List of Essential Medicines.

[22] Lai C.K.W., Beasley R., Crane J., Foliaki S., Shah J., Weiland S., the ISAAC Phase Three Study Group (2009). Global variation in the prevalence and severity of asthma symptoms: Phase Three of the International Study of Asthma and Allergies in Childhood (ISAAC). Thorax.

[23] World Bank World Bank Country and Lending Groups. Country Classification 2014. https://datahelpdesk.worldbank.org/knowledgebase/.

[24] Perehudoff S.K., Alexandrov N.V., Hogerzeil H.V. (2018). Access to essential medicines in 195 countries: A human rights approach to sustainable development. Glob. Public Health.

[25] Perehudoff S.K., Laing R.O., Hogerzeil H.V. (2010). Access to essential medicines in national constitutions. Bull. World Health Organ..

[26] Laing R., Waning B., Gray A., Ford N., t Hoen E. (2003). 25 years of the WHO essential medicines lists: Progress and challenges. Lancet.

[27] United Nations Sustainable Development Goals 2015. https://sustainabledevelopment.un.org/sdgs.

[28] World Health Organization (2010). Key Components of a Well Functioning Health System.

[29] Gray A.L., Wirtz V.J., t Hoen E.F., Reich M.R., Hogerzeil H.V. (2015). Essential medicines are still essential. Lancet.

[30] World Health Organization (2015). WHO Moves to Improve Access to Lifesaving Medicines for Hepatitis C, Drug-Resistant TB and Cancers [Press Release].

[31] Bigdeli M., Jacobs B., Tomson G., Laing R., Ghaffar A., Dujardin B., Van Damme W. (2013). Access to medicines from a health system perspective. Health Policy Plan..

[32] Ade G., Gninafon M., Tawo L., Ait-Khaled N., Enarson D.A., Chiang C.-Y. (2013). Management of asthma in Benin: The challenge of loss to follow-up. Public Health Action..

[33] El Sony A.I., Chiang C.-Y., Malik E., Hassanain S.A., Hussein H., Khamis A.H., Bassilli A.F., Enarson D.A. (2013). Standard case management of asthma in Sudan: A pilot project. Public Health Action..

[34] Kan X.H., Chiang C.-Y., Enarson D.A., Rao H.L., Chen Q., Ait-Khaled N. (2012). Asthma as a hidden disease in rural China: Opportunities and challenges of standard case management. Public Health Action.

[35] Frost L.J., Reich M.R. (2008). Access: How Do Good Health Technologies Get to Poor People in Poor Countries.

[36] Davidsen J.R., Sondergaard J., Hallas J., Siersted H.C., Knudsen T.B., Lykkegaard J., Andersen M. (2011). Impact of socioeconomic status on the use of inhaled corticosteroids in young adult asthmatics. Respir. Med..

[37] Duong M., Moles R.J., Chaar B., Chen T.F., World Hospital Pharmacy Research Consortium (2015). Essential Medicines in a High Income Country: Essential to Whom?. PLoS ONE.

[38] Taglione M.S., Ahmad H., Slater M., Aliarzadeh B., Glazier R.H., Laupacis A., Persaud N. (2017). Development of a Preliminary Essential Medicines List for Canada. CMAJ Open.

[39] Eom G., Grootendorst P., Duffin J. (2016). The case for an essential medicines list for Canada. CMAJ.

[40] Pearce N., Asher I., Billo N., Bissell K., Ellwood P., El Sony A., García-Marcos L., Chiang C.-Y., Mallol J., Marks G. (2013). Asthma in the global NCD agenda: A neglected epidemic. Lancet Respir. Med..

[41] Asher I., Bissell K., Chiang C.-Y., El Sony A., Ellwood P., García-Marcos L., Marks G.B., Mortimer K., Pearce N., Strachan D. (2019). Calling time on asthma deaths in tropical regions-how much longer must people wait for essential medicines?. Lancet Respir. Med..

[42] Robertson J., Macé C., Forte G., de Joncheere K., Beran D. (2015). Medicines availability for non-communicable diseases: The case for standardized monitoring. Glob. Health.

[43] Wirtz V.J., Hogerzeil H.V., Gray A.L., Bigdeli M., de Joncheere C.P., Ewen M.A., Gyansa-Lutterodt M., Jing S., Luiza V.L., Mbindyo R.M. (2017). Essential medicines for universal health coverage. Lancet.

[44] Enarson D.A. (2005). Fostering a spirit of critical thinking: The ISAAC story. Int. J. Tuberc. Lung Dis..

[45] World Health Organization (2008). Measuring Medicine Prices, Availability, Affordability and Price Components.

